# Addressing Social Determinants of Health from the Emergency Department through Social Emergency Medicine

**DOI:** 10.5811/westjem.2016.5.30240

**Published:** 2016-06-21

**Authors:** Erik S. Anderson, Suzanne Lippert, Jennifer Newberry, Edward Bernstein, Harrison J. Alter, Nancy E. Wang

**Affiliations:** * Highland Hospital, Alameda Health System, Department of Emergency Medicine, Oakland, California; † Stanford University, Department of Emergency Medicine, Stanford, California; ‡ Boston University, Department of Emergency Medicine, Boston, Massachusetts

Dialogue and policy surrounding healthcare reform have drawn increasing interest to the social factors, accountable for nearly one-third of annual deaths in the United States,^1^ that affect the health of populations. The Affordable Care Act (ACA) includes provisions for health systems to address social determinants of health, but how this is to be accomplished remains uncertain. If we are to make progress as a health system in addressing social determinants of health, we must open a dialogue and practice that reaches patients at the front lines of the medical system and population health – including in the emergency department (ED). The fact that emergency physicians care for patients who are complicated both medically and socially is no surprise, but the idea that we have an important role to play in the social determinants of health of our patients is, while controversial, gaining increasing attention among emergency physicians across the country. This interest comes largely from necessity, as we face a daunting task of providing care to the large volume of vulnerable patients who seek refuge in our EDs.

The ED is a window into the community, which starkly frames the contributions of the social determinants underlying the trauma resuscitations, repeat child visits for asthma exacerbation, or sepsis due to delay in seeking care. In the ED, we diagnose and treat the medical problem – but in order to improve the health of our patients we need to expand our role to diagnose and treat their social determinants of health as well. We urge our colleagues to not only consider the social determinants underlying health and illness, but to also develop systematic interventions, measure their effects, collaborate with others, and advocate for policies that will improve the health of our patients. We advocate physicians to address the social determinants of health from the ED, in other words, to practice Social Emergency Medicine.

The World Health Organization defines the social determinants of health as “conditions in which people are born, grow, live, work, and age. These circumstances are shaped by the distribution of money, power and resources at global, national and local levels.”^2^ It seems obvious that poverty, racial and ethnic inequities, and lack of preventive care, would lead to poor health. But the social determinants of health extend beyond these more tangible aspects of our lives. Every aspect of how we live, including social class, influences health profoundly. Even among London-based British civil servants leading relatively stable lives with guaranteed employment, salary and health insurance, there is a steep and inverse correlation between job classification level, morbidity and death.^3^ From a policy standpoint, this gradient is compelling, as it affects all of our patients, not just those living in poverty, but the middle class as well.

Given that the structure of our daily lives are the social determinants of health, doing something about them requires moving our focus from the single patient to the population level, from diagnostics and medications to environmental and social structures and the policies that create them. While it would be clear to most emergency physicians that a patient’s frequent visits for hyperglycemia reflect poorly managed diabetes, what is easily labeled willful noncompliance might instead be a lack of access to healthy foods, and ultimately insufficient social and technical support for the entire community. Thus, medical treatment of a disease such as diabetes, without regard to the social determinants of health, suffers the danger of being ineffective. Just as we cannot treat volume overload without understanding the physiology of the kidney, heart, lungs and their interaction, we cannot begin to treat a patient’s medical problems without understanding the social factors, the life he lives.

Necessity mandates action. While the ACA tasks primary care with managing these social determinants, access to medical care increasingly occurs through the ED for insured, as well as poor and marginalized populations.^4^ The ED is the only door open to anyone for comprehensive medical and social services, 24 hours a day, 7 days a week, regardless of acuity or complaint, age, or insurance status. The status of the ED as society’s “safety net” is reinforced by a legal imperative, embodied in the Emergency Medical Treatment and Labor Act of 1986, which requires Medicare-participating hospitals offering emergency services to provide a medical screening examination and stabilization of emergency conditions regardless of ability to pay. What we face practicing in this safety net is an imperative to act. We must embrace this role and adopt our practice to our *de facto* environment, as a critical part of out healthcare safety net. Applying knowledge about social determinants of health to the bedside and developing effective, systematic interventions that reach out into the community is the practice of Social Emergency Medicine.

With increasing ED volumes and ED crowding in the headlines, some argue that taking on this burden would interfere with the ED’s primary mission of caring for the acute and emergent medical problems of the patients, and only when funded appropriately, should EDs take on this mammoth task. However, practically speaking, patients inadequately treated will continue to return to the ED. Many EDs already screen for vulnerable patients and offer some preventive services. ED directors are not philosophically opposed to offering these services within the ED, but are concerned with added costs, effects on ED operations, and potential lack of follow up.^5^ We believe that to ignore the contribution of social determinants on disease simply because addressing them requires unbudgeted resources, including sophisticated coordination of clinical, statistical, social and policy expertise, is as great an omission as ignoring the contribution of genetics simply because we do not yet have the tools to reliably control gene expression.

EDs are beginning to take ownership of social determinants of health for their patients. Recent examples of successful Social Emergency Medicine interventions have focused on the development of coordinated care models providing ED patients in need with comprehensive medical and social services. Emergency medicine researchers worked with the Housing First partnership between the Centers for Medicare and Medicaid Services and New York City, which provided housing for high-risk homeless patients, resulting in improved health and cost savings for the city.^6^ Boston Medical Center has a robust youth violence intervention program integrated into ED clinical care.^7^ Emergency medicine has advocated for policies and programs to improve the care of patients with substance use disorders such as implementing screening, brief intervention, and referral to treatment programs and providing take-home naloxone to prevent opioid overdose.^8^,^9^

A fundamental step towards making the practice of Social Emergency Medicine more feasible requires integrating the study of the social determinants of health into our education. Medical training in the social determinants cannot be relegated to a single lecture or seminar, but rather requires a proportional emphasis along with anatomy, pharmacology and pathophysiology of disease. Similarly, we must not only teach the relationship of social determinants and health, but also teach the tools to translate theory into practice. We should teach methods to collaborate with community groups and design interventions so that young doctors do not segregate their medical and social diagnoses and interventions.

A fitting consequence of developing a subspecialty of Social Emergency Medicine would be that while all medical practitioners must know some theory, basic diagnostics and treatment; complicated cases require expert consultation and a systemwide effort. A single physician recognizing that a patient’s unstable housing is an impediment to proper management of his health is important, but the next steps can feel daunting – especially in the face of a full waiting room and critically ill patients. This burden cannot fall on the individual clinician; isolated interventions will fail. Although a physician can recognize that her patient is suffering an ST elevation myocardial infarction, she requires a system to achieve timely medical and procedural intervention resulting in favorable outcomes. Accordingly, successful Social Emergency Medicine interventions require specialty training, resources, and a multidisciplinary team.

Physicians practicing Social Emergency Medicine must also network, establish, and foster collaborations. Screening programs and innovative interventions cannot be solely well intentioned, but must be needs based and proven effective. Sharing of resources, best practices, standardization of data collection, and research networks with the dissemination of findings are imperative. Social Emergency Medicine initiatives should culminate in advocacy for policies to combat the adverse health impacts that stem from the vastly disparate conditions in which people are born, grow, live, work, and age.

One can view the ED (by law, the most accessible door into our healthcare system) as the social barometer of its community. Within the waiting room the emergency physicians witness the confluence of social determinants of health and their deconstruction into pathology. Our daily practice compels us to act, to systematically and collaboratively act on upstream social factors to positively and comprehensively influence downstream health outcomes. This paradigm shift is critical to effectively care for our patients. In the words of Rudolph Virchow, “Medicine has imperceptibly led us into the social field and placed us in a position of confronting directly the great problems of our time.”^10^
